# Supplementary material for: Association of maternal obesity with fetal and neonatal death: Evidence from South and South-East Asian countries
Source: PLoS One. 2021 Sep 2;16(9):e0256725. doi: 10.1371/journal.pone.0256725 (PMC8412251; doi:10.1371/journal.pone.0256725)
Supplement: Attachment — Submitted filename: Response to the Reviewers.docx [file pone.0256725.s001.docx]

**Response to the Editor’s comments**

| **Editor’s Comments** | | | |
| --- | --- | --- | --- |
| **Editor’s Comments** | | **Authors’ response** | **References** |
| 1 | Table 1 includes both descriptive and inferential (95% CIs) statistics. Given that the aim is not to estimate the prevalence of participants with different characteristics in the population, it is not useful to present the 95% CIs. It is more meaningful to show the distribution of the potential confounding variables (rows) according to the exposure variable (column). This will help in assessing for imbalances in the distribution of the confounders. | The authors would like to thank the reviewer for this helpful comment. We excluded the 95% CI values from Table 1. In Table 2, we presented the results of the bivariate analysis to show the distribution of outcome variables (column) with exposure and potential confounding variables (row). | Changes are highlighted in red coloured text in the revised manuscript. Please refer to the “estimation strategies” section and to Table 1.  Page 7, lines 10-12 and  Page 9 (Table 1) |
| 2 | It is necessary to properly account for the sampling weights when pooling data across surveys. It is not clear how sampling weights were handled in this paper. This is useful reference when dealing with weights when analysing DHS data Multilevel Modeling Using DHS Surveys: A Framework to Approximate Level-Weights [MR27] (dhsprogram.com) | The authors express thanks to the Editor for this suggestion. Sampling weights are now incorporated in the analysis and mentioned clearly in the estimation strategies as follows:    “The DHS data involve multistage sampling, unequal sampling probabilities, and stratification. Therefore, before performing any statistical analysis, this study weighted the data using sampling weights, primary sampling unit, strata and cluster to restore the representatives of the survey and futher to calculate standard errors and reliable estimates as per the DHS framework to approximate level-weights [1]. STATA command “svy set” was used for this analysis to address the complex survey design. All analyses such as summary statistics, cross-tabulation and regression were conducted using the STATA software, version 14”. | Changes are highlighted in red coloured text in the revised manuscript. Please refer to the “estimation strategies” section  Page 7, lines 4-10 |
| 3 | It is also unclear whether other features of the DHS survey i.e. strata and clusters, were properly accounted for in the analysis. | Thank you for your comment. Strata, clusters and other features of the DHS survey are accounted for in the analyses; the revised text reads as follows:  “The DHS data involve multistage sampling, unequal sampling probabilities, and stratification. Therefore, before performing any statistical analysis, this study weighted the data using sampling weights, primary sampling unit, strata and cluster to restore the representatives of the survey and futher to calculate standard errors and reliable estimates as per the DHS framework to approximate level-weights [1]. STATA command “svy set” was used for this analysis to address the complex survey design. All analyses such as summary statistics, cross-tabulation and regression were conducted using the STATA software, version 14”. | Marked in red text in the paper. Please refer to the “estimation strategies” section  Page 7, lines 4-10 |
| 4 | Was multi-level analysis considered? | We agree with the Editor and thus performed an additional multi-level analysis. The revised text reads as follows:  “Since the DHS data has a hierarchical structure, in this study, multivariable multilevel logistic regression analysis was also applied to determine the effects of each independent variable of miscarriage, stillbirth, early neonatal mortality and late neonatal mortality. The multilevel analysis was conducted separately in two regression models for each outcome variables: miscarriage, stillbirth, early neonatal mortality and late neonatal mortality. Model 1 represented a null model was fitted without independent variables to test random variability in the intercept. Null model also used to estimate the intra-class correlation coefficient (ICC). Model 2, a mixed model, included all independent variables which were found to be statistically significant (<0.05) in the bivariate analysis, was examined the effects of all considered independent variables simultaneously.  This study estimated both fixed and random effect parameters from multivariate multilevel modelling. The fixed and random effect models measured the adjusted odds ratios and country-level variance, respectively. Furthermore, Intraclass correlation coefficient (ICC), calculating the variance in percentage explained by the country-level factors, were used to measure the random effects”. | Marked with red text in the paper. Please refer to the “estimation strategies” section.  Page 8, lines 4-13 |
| 5 | Please present both unadjusted and adjusted ORs. | Thank you for your advice. Both adjusted and unadjusted ORs are now included. | Please refer to Table 3 in  Page 13 |
| 6 | In Table 3, it is unnecessary to present ORs for potential confounders. | Thank you for your suggestion. The authors agreed and omitted the potential confounders. Now, Table 3 only reported the exposure variables. | Please refer to Table 3 in  Page 13 |
| 7 | Did you explore for any interactions? | Yes, we explored the interactions of exposure variable of mother’s BMI with potential confounding variables of age, area of residence, and mother’s education for four outcome variables (e.g., miscarriage, stillbirth, early neonatal mortality and late neonatal mortality). However, no statistically significant associations were found. Therefore, results are not reported in the paper.  This has been stated in the revised manuscript. | Please see the appendix provided at the bottom of this file “Response to reviewer” for the results of interactions, pages 15-23.  For the revised text, see page 8, lines 19-22. |
| 8 | In the tables, it is not easy to visually distinguish between variables their categories. | Thank you very much for pointing this out. The variables and categories are now made clearly visible. | Please refer to table 1, 2, 3 and 4.  Pages 9-10  Pages 11-12  Page 13  Pages 15 |

**Response to the reviewer’s comments**

| **Reviewer-I** | | | |
| --- | --- | --- | --- |
| **Reviewer’s comments** | | **Authors’ response** | **References** |
| Thank you for the opportunity to contribute to the peer review process for this research. The study aims to analyse the association between maternal obesity and adverse pregnancy outcome and neonatal death in South and South-East Asian countries. The study is interesting and provides novel data on the association in South and South-East Asian Countries. However, this paper has some points that need to be clarified: | | Thank you very much for the appreciation. The authors are grateful to the reviewer for the insightful feedack. | Not Applicable (N/A) |
| 1. | In Methodology section, authors should indicate which was the statistical package that they used for data analysis | Thank you for your comments. The authors now mentioned the statistical package used for data analysis as follows:  “All analyses such as summary statistics, cross-tabulation and regression were conducted using the STATA software, version 14”. | Marked with red text in the paper. Please refer the “estimation strategies” section  Page 7, lines 9-10 |
| 2. | In Methodology section (line 106), the authors say “This research selected 8 out of 15 countries in South and South-East Asia..”. I suggest to the authors indicate which were the countries that they included in the study. | Thank you very much for your suggestion. The authors now mentioned the countries included in this analysis as follows:  “This research selected and pooled the most recent surveys from eight out of 15 countries of South and South-East Asia available in the DHS database. These countries include Cambodia (2014), East Timur (2014), India (2015-16), Myanmar (2015-16), Nepal (2016), Maldives (2016-17), Bangladesh (2017-18) and Pakistan (2017-18).” | Marked with red text in the paper. Please refer the “Study participants” section  Page 5, lines 5-9 |
| 3. | Lines 134-144, the explanation about the BMI is confusing. The authors mention the BMI categorisation according to the WHO authors: “Maternal weight status was the exposure of interest, and it was ascertained through the mother’s BMI. BMI was calculated as weight in kg divided by the height in m2, and it was categorised into four groups following the WHO guidelines, namely, underweight (BMI <18.50), healthy weight (BMI 18.50 to <25.00), overweight (BMI 25.00 to <30.00) and obese (BMI ≥30) (2)”. And after that they say “Since the targeted population of this study was South and South-East Asia, we used the cut off points of BMI for Asians, which are categorised as underweight (BMI< 18.50), healthy weight (BMI 18.50 to 22.90), overweight (BMI 23 to 26.9) and obese (BMI> 27) (19)”. If the authors used the BMI for Asians categorisation, it is unnecessary to indicate the WHO BMI scores. Simply naming it, I think it is enough, and it makes the text easier to understand. It would be necessary to add the WHO reference. | Thank you for pointing this out. The authors agree with the reviewer and deleted the WHO BMI guidelines to avoid confusions. The text is now written as:  “BMI was calculated as the ratio of weight in kilograms (kg) divided by height in metres-squared (m^2^), and it was categorised into four groups following the WHO guidelines [2]. Given that the target population of this study was drawn from South and South-East Asia, we used the cut off points of BMI for Asians, which are categorised as underweight (BMI< 18.50), healthy weight (BMI 18.50 to 22.90), overweight (BMI 23 to 26.9) and obese (BMI> 27) [3]. The reason for using a separate BMI calculator for Asian and less-developed countries has been discussed in prior studies where the authors considered ethnical diversity that results in small body size and lower gestational weight gain compared to developed countries [4,5]” | Marked with red text in the paper. Please refer the “Exposure variable” section  Page 6, lines 10-17 |
| 4. | The authors indicate in the abstract the range of years from which they collected data for the study. However this information is missing in Methodology section. I suggest that the authors also indicate this information in the text (and not only in figure 1). | Thank you for your advice. Years of data collection for each country are now written in the methodology section as follows:  “This research selected and pooled the most recent surveys from eight out of 15 countries of South and South-East Asia available in the DHS database. These countries include Cambodia (2014), East Timur (2014), India (2015-16), Myanmar (2015-16), Nepal (2016), Maldives (2016-17), Bangladesh (2017-18) and Pakistan (2017-18).” | Marked with red text in the paper. Please refer the “Study participants” section  Page 5, lines 5-9 |
| 5. | Introduction, lines 73-76. In order to unify the referenced format, please remove the year in parentheses and indicate the reference number immediately after each author. | Thank you for your suggestion. The referencing style has been updated for the whole manuscript. Now the text has been revised as:  “Baroni et al. [6] investigated the neonatal mortality rates in Brazil, whereas Liu et al. [7] and Abdul-Mumin et al. [8] investigated the key reasons for neonatal death and preterm birth in China and Ghana”. | Marked with red text in the paper. Please refer the “Introduction section”.  Page 3, lines 19-21 |
| 6. | In Methodology section, lines 103-105, the authors say “The study pooled the most recent survey in eight South and South-East Asian countries”. Again, in line 107 they say “This research selected 8 out of 15 countries in South and South-East Asia”. From my point of view this information is redundant. I suggest to the authors unify this information in the same sentence/paragraph. | Thank you very much for pointing this out. To avoid the redundancy, the authors deleted lines 103-105 in Methodology section and now unify this information in the same sentence in the study participants as follows:  “This research selected and pooled the most recent surveys from eight out of 15 countries of South and South-East Asia available in the DHS database. These countries include Cambodia (2014), East Timur (2014), India (2015-16), Myanmar (2015-16), Nepal (2016), Maldives (2016-17), Bangladesh (2017-18) and Pakistan (2017-18).” | Marked with red text in the paper. Please refer the “Study participants” section  Page 5, lines 5-9 |
| 7. | Authors should use past tense in the manuscript. For example, line 108 “The remaing seven countries are excluded..” should be written “were”. Please, check it in the whole text. | We have rewritten the sentence in past tense and also checked for consistency in the whole paper. The sentence is re-written as:  “The remaining seven countries were excluded from the analysis due to publicly inaccessible, inadequate and/or obsolete data”. | Marked with red text in the paper. Please refer the “Study participants” section  Page 5, lines 8-9 |
| 8. | In References section, the reference 8 is incomplete. | Thank you very much for raising this issue. Reference 8 is now written in appropriate style as follows:  Liu Y, Kang L, He C, Miao L, Qiu X, Xia W, et al. Neonatal mortality and leading causes of deaths: a descriptive study in China, 2014–2018. 2021;11:e042654. doi:10.1136/bmjopen-2020-042654 | Marked with red text in the paper. Please refer the “Reference” section  Page 21, reference 8 |
| Reviewer-II | | | |
| **Reviewer’s comments** | | **Authors’ response** | **References** |
| The study used pooled data from the most recent DHS survey from 8 South and South-East Asian countries. Multivariate logistic regression was deployed to check the relationships between maternal obesity and fetal deaths. It is found that maternal obesity associated with a greater risk of perinatal and early neonatal deaths. Overall this paper addresses the important issues. However, a number of improvements are needed. Some key points are listed below: | | We thank the Reviewer for appreciating our work and constructive comments and suggestions. | Not Applicable (N/A) |
| 1. | In this study, authors sometime mention perinatal and neonatal death, sometime adverse reproductive health outcome, and sometime foetal death. It should be consistence. Also, what is the meaning of foetal death? | The terminologies have been synchronised for consistency. Both miscarriage and stillbirth are referred to as fatal death. Mentioning “foetal death” was a mistake which has now been corrected. | Marked with red text in the paper. For example  Please refer to  Page 2, lines 3,4, 22  Page 3, line 15  Page 5, lines 18,19  Page 9, line 1  Page 13, line 1  Page 16, line 2  Page 17, line 25  Page 18, line 8  Page 19, lines 6,8 |
| 2. | In the first paragraph of the introduction, authors told about adults overweight and obese. The authors should give the world statistics for maternal obese. | World statistics for women have been provided for overweight and obese. It is now written as:  “Globally, it was estimated that 40% and 15% of women aged 18 years and above were overweight and obese, respectively, in 2016 [2]” | Marked with red text in the paper. Please refer the “Introduction” section  Page 3, lines 8-10 |
| 3. | In line 74-76 authors identify the key reasons for neonatal death but didn’t mention the reasons “Baroni et al. (2021) investigated the neonatal mortality rates in Brazil, whereas Liu et al. (2020) and Abdul-Mumin et al. (2021) investigated the key reasons for neonatal death in China and Ghana, respectively, including preterm birth (7–9)” | The study in Brazil has investigated the neonatal rate only. Hence, the reasons for neonatal deaths in China and Ghana have been mentioned as:  “Regional investigations have identified that preterm birth, intrapartum complications and pneumonia (in case of China), and preterm birth complications and birth asphyxia (in case of Ghana) are the leading causes of neonatal deaths [7,8]” | Marked with red text in the paper. Please refer the “Introduction” section  Page 3, lines 21-24 |
| 4. | This study used recent pooled data from 8 South and South-East Asian countries but for Bangladesh this study used old dataset. | Thank you very much for focusing this issue. The recent DHS data for Bangladesh 2017-18 is now used in this study. All the analyses are re-estimated and presented in Tables 1, 2,3 and 4. | Marked with red text in the paper. Please refer the “Study participants” section. Please also see Tables 1, 2 and 3 for updated results.  Page 5, line 8  Please refer to table 1, 2, 3 and 4.  Pages 9-10  Pages 11-12  Page 13  Pages 15 |
| 5. | This study used multivariable logistic regression model to explore the association between exposure and outcome. But all the outcome proportion less than 10% better to use Poisson regression. Moreover, this study used multi-country data, so there is some country level variation for each outcome. For the multi-country data better to use multi-level approach. | Thank you for your suggestion. Poisson Regression models are best used for modelling events where the outcomes are counts [9]. Given that, all of our outcome variables are categorical, we used logistic regression.  As the reviewer suggests, the authors conducted the multi-level analysis to see the country-level variations for each outcome; the revised text reads as follows:  “Since the DHS data has a hierarchical structure, in this study, multivariable multilevel logistic regression analysis was also applied to determine the effects of each independent variable of miscarriage, stillbirth, early neonatal mortality and late neonatal mortality. The multilevel analysis was conducted separately in two regression models for each outcome variables: miscarriage, stillbirth, early neonatal mortality and late neonatal mortality. Model 1 represented a null model was fitted without independent variables to test random variability in the intercept. Null model also used to estimate the intra-class correlation coefficient (ICC). Model 2, a mixed model, included all independent variables which were found to be statistically significant (<0.05) in the bivariate analysis, was examined the effects of all considered independent variables simultaneously.  This study estimated both fixed and random effect parameters from multivariate multilevel modelling. The fixed and random effect models measured the adjusted odds ratios and country-level variance, respectively. Furthermore, Intraclass correlation coefficient (ICC), calculating the variance in percentage explained by the country-level factors, were used to measure the random effects” | Marked with red text in the paper. Please refer the “Estimation strategies” section.  Page 8, lines 4-18 |
| 6. | This study used some upper-middle income country and some lower-middle income countries. Authors should preform sensitivity analysis. | Thank you for your advice. Given that the authors performed a multi-level analysis, sensitivity is already revealed as follows:  “The null model (Model 1) of miscarriage described that significant variation exists in the odds across the countries (τ=0.72; 95% CI: 0.44 - 1.18; p<0.001). The variance estimate was greater than zero, it indicates that there were country differences in miscarriage among women in south and south-east Asia, and thus multilevel analysis should be considered as an appropriate approach for further analysis. Likewise, after controlling the effect of independent variables at the country level, the variance at the country level had a significant impact (τ=0.70; 95% CI: 0.43- 1.15; p<0.001) in Model 2. The null model (Model 1) demonstrated that, overall, 13.59% variation in the odds of miscarriage was reported that involved cluster difference of the characteristics (ICC=13.59%). The variation between clusters declined to 12.92% in Model 2. The result of Model 2 exposed that the adjusted odds of miscarriage among obese mothers were 1.19 (aOR: 1.19; 95% CI: 1.13-1.26) times higher compared to mothers with a healthy weight.  Concerning the second outcome variable, stillbirth, both the null model (Model 1) and mixed model (Model 2) confirmed significant variation (variance estimate greater than zero) in the odds across the country (τ=1.01; 95% CI: 0.60-1.69; p<0.001 and τ=0.99; 95% CI: 0.58-1.66; p<0.001 respectively). The variation of odds between the clusters declined from 23.73% to 22.95% from Model 1 to Model 2 (ICC=23.73% and ICC=22.95%, respectively). The result from the mixed-model showed that mothers with obesity were more likely to report stillbirth (aOR:1.31, 95% CI: 1.14-1.51) compared with those with a healthy BMI range.  In the case of early neonatal mortality, variation in the odds across the country was zero which confirms that there were country differences in early neonatal mortality among women in south and south-east Asia. The variance at the country level reduced from null model (τ=0.39; 95% CI: 0.23-0.57; p<0.001) to mixed model (τ=0.37; 95% CI: 0.21-0.64; p<0.001). The variation of odds between the clusters declined from 4.42% to 3.96% from Model 1 to Model 2. The mixed model results revealed that the mothers with obesity had elevated odds (aOR: 1.20, 95% CI: 1.10-1.31) of having early neonatal death compared with mothers possessing healthy weight. However, no significant association was found in the mixed model between maternal obesity and late neonatal death”. | Marked with red text in the paper. Please refer the “Results section”.  Page 14, lines 1-31 |
| 7. | In the abstract conclusion, authors mention “Obese women need to follow antenatal care visits and deliver at the institutional facility centre that can offer obstetrics and early neonatal care” but they didn’t discuss in the main conclusion. Conclusion should be consistence. | We have rewritten the section to ensure consistency. In conclusion, now it has been written as:  “Besides, antenatal care visits by obese women and delivery at a centre offering services by specialist obstetrics and dedicated early neonatal care should also be ensured.”. | Marked with red text in the paper. Please refer the “Conclusion section”.  Page 19, lines 10-12 |
| 8. | In the conclusion, authors mention “An effective intervention to maintain recommended weight and lifestyle choices of mother is needed to reduce the number of cases of stillbirth, miscarriage and neonatal death. Moreover, cost-effective community-based strategies to improve maternal education are required in this region to achieve SDG Target-3, which aims to ensure healthy lives and promote the well-being of people of all ages by 2030”. It would be better if the authors mention what kind of effective intervention and cost-effective community-based strategies are needed? | Since we have rewritten the section, specific interventions, like antenatal care visits and delivery at dedicated center have been incorporated in the revised manuscript. Cost-effective community-based strategies such as nutrition program, volunteering for immediate support, and maternal education have also been recommended. | Marked with red text in the paper. Please refer the “Conclusion section”.  Page 19, lines 12-14 |
| 9. | I found several mistakes in references styles. If the reference has doi and volume number don’t need to write [Internet] and available from. Please follow the journal reference style. | We have corrected all the references according to the journal reference style guidelines and also ensured their consistency. | Marked with red text in the paper. Please refer the “Reference” section  Page 21-24, Reference 1-43 |
| 10. | Reference number 8 incomplete. | Thank you very much for highlighting this issue. The reference 8 is now written in appropriate style as:  Liu Y, Kang L, He C, Miao L, Qiu X, Xia W, et al. Neonatal mortality and leading causes of deaths: a descriptive study in China, 2014–2018. 2021;11:e042654. doi:10.1136/bmjopen-2020-042654 | Marked with red text in the paper. Please refer the “Reference” section  Page 21, reference 8 |

**References:**

1.	Elkasabi M, Ren R, Pullum TW. Multilevel Modeling Using Dhs Surveys: a Framework To Approximate Level-Weights. DHS Methodol Reports No 27. Rockville, Maryland, USA: ICF; 2020.

2.	Obesity and Overweight. In: World Health Organization [Internet]. [cited 13 Feb 2021]. Available: https://www.who.int/news-room/fact-sheets/detail/obesity-and-overweight

3.	Asian BMI Calculator. In: Joslin Diabetes Center [Internet]. [cited 25 Feb 2021]. Available: https://aadi.joslin.org/en/am-i-at-risk/asian-bmi-calculator?fbclid=IwAR1yhCxSOjS0jeEOCnvdh_orXAPJmoCBmOTCWJdLzy7ompm_X781HNxMUeg

4.	Ota E, Haruna M, Suzuki M, Anh D, Tho H, Thanh T. Maternal body mass index and gestational weight gain and their association with perinatal outcomes in Viet Nam. 2011; 127–136. doi:10.2471/BLT.10.077982

5.	Gondwe A, Ashorn P, Ashorn U, Dewey KG, Maleta K, Nkhoma M, et al. Pre-pregnancy body mass index (BMI) and maternal gestational weight gain are positively associated with birth outcomes in rural Malawi. PLoS One. 2018;13: 1–15. doi:10.1371/journal.pone.0206035

6.	Baroni L, Salles R, Salles S, Pedroso M, Lima J, Morais I, et al. Neonatal mortality rates in Brazilian municipalities : from 1996 to 2017. BMC Res Notes. 2021; 20–22. doi:10.1186/s13104-020-05441-3

7.	Liu Y, Kang L, He C, Miao L, Qiu X, Xia W, et al. Neonatal mortality and leading causes of deaths: a descriptive study in China, 2014–2018. 2021;11:e042654. doi:10.1136/bmjopen-2020-042654

8.	Abdul-Mumin A, Cotache-Condor C, Owusu SA, Mahama H, Smith ER. Timing and causes of neonatal mortality in Tamale Teaching Hospital, Ghana: A retrospective study. PLoS One. 2021;16: 1–12. doi:10.1371/journal.pone.0245065

9.	Hutchinson MK, Holtman MC. Analysis of count data using Poisson regression. Res Nurs Heal. 2005;28: 408–418. doi:10.1002/nur.20093

**Table 4. Multivariate Multi-level logistic regression for fetal and neonatal death**

|  | **Miscarriage** | | **Stillbirth** | |
| --- | --- | --- | --- | --- |
|  | Model 1 | Model 2 | Model 1 | Model 2 |
| **Exposure Variable** |  |  |  |  |
| Mothers’ BMI |  |  |  |  |
| *Underweight* |  | 0.97 (0.93-1.01) |  | 0.97 (0.88-1.07) |
| *Healthy weight (ref)* |  |  |  |  |
| *Overweight* |  | 1.06**(1.02-1.11) |  | 1.03 (0.93-1.15) |
| *Obesity* |  | 1.19***(1.13-1.26) |  | 1.31*** (1.14-1.51) |
| **Intercept** | 0.11*** (0.07-0.19) | 0.07*** ( 0.04-0.12) | 0.01*** (0.01-0.02) | 0.01***(0.01-0.02) |
| **Model summary (Random Effect)** |  |  |  |  |
| Country Variance | 0.72 (0.44-1.18) | 0.70 (0.43-1.15) | 1.01 (0.60-1.69) | 0.99 (0.58-1.66) |
| LR test | P <0.001 | P <0.001 | P <0.001 | P <0.001 |
| ICC (%) | 13.59% | 12.92% | 23.73% | 22.95% |
| Log likelihood | -58738.35 | -58201.658 | -13688.088 | -13464.331 |
| AIC | 117480.7 | 116459.3 | 27380.18 | 26984.66 |
|  |  |  |  |  |
|  | **Early neonatal mortality** | | **Late neonatal mortality** | |
|  | Model 1 | Model 2 | Model 1 | Model 2 |
| **Exposure Variable** |  |  |  |  |
| Mothers’ BMI |  |  |  |  |
| *Underweight* |  | 1.07* (1.01-1.13) |  | 1.05 (0.93-1.19) |
| *Healthy weight (ref)* |  |  |  |  |
| *Overweight* |  | 1.02 (0.96-1.09) |  | 1.04 (0.90-1.21) |
| *Obesity* |  | 1.20*** (1.10-1.31) |  | 1.13 (0.92-1.39) |
| **Intercept** | 0.04*** (0.03-0.05) | 0.01***(0.01-0.02) | 0.01***(0.01-0.02) | 0.01***(0.01-0.02) |
| **Model summary (Random Effect)** |  |  |  |  |
| Country Variance | 0.39 (0.23-0.57) | 0.37 (0.21-0.64) | 0.57 (0.31-1.05) | 0.50 (0.26-0.96) |
| LR test | P <0.001 | P <0.001 | P <0.001 | P <0.001 |
| ICC (%) | 4.42% | 3.96% | 9.06% | 6.98% |
| Log likelihood | -35471.749 | -32027.656 | -8718.8771 | -8278.1581 |
| AIC | 70947.5 | 64111.31 | 17441.75 | 16612.32 |

P-values: ***P < 0.001, **P < 0.01, * P< 0.05

Model 1: No covariates controlled for.

Model 2: Only exposure variables are reported. The model adjusted with age, education, employment status, wealth index, civil status, area of residence, age at first marriage, number of children, age at first birth, and place of delivery.

**Appendix 1: Interactions of exposure variable mother’s BMI with potential confounding variables age, area of residence mother’s education for four outcome variables (miscarriage, stillbirth, early neonatal mortality and late neonatal mortality)**

**Figure 1: Miscarriage and interaction with Mother’s BMI and age**

**Figure 2: Miscarriage and interaction with Mother’s BMI and area of residence**

**Figure 3: Miscarriage and interaction with Mother’s BMI and mother’s education**

**Figure 4: Stillbirth and interaction with Mother’s BMI and age**

**Figure 5: Stillbirth and interaction with Mother’s BMI and area of residence**

**Figure 6: Stillbirth and interaction with Mother’s BMI and mother’s education**

**Figure 7: Early neonatal mortality and interaction with Mother’s BMI and age**

**Figure 8: Early neonatal mortality and interaction with Mother’s BMI and area of residence**

**Figure 9: Early neonatal mortality and interaction with Mother’s BMI and mother’s education**
