# Supplementary material for: Association of maternal obesity with fetal and neonatal death: Evidence from South and South-East Asian countries
Source: PLoS One. 2021 Sep 2;16(9):e0256725. doi: 10.1371/journal.pone.0256725 (PMC8412251; doi:10.1371/journal.pone.0256725)
Supplement: Attachment — Submitted filename: Response to the Reviewers.docx [file pone.0256725.s002.docx]

**Response to the reviewer’s comments**

| Reviewer-II | | | |
| --- | --- | --- | --- |
| **Reviewer’s comments** | | **Authors’ response** | **References** |
| 1. | You have pooled data from 8 countries. Where is weighting? I mean your data weighting after pooling as you deal with multiple countries with wide variation in their population? E.g. India have 87.05% population (Figure 1). Could you please explain more detail about weight? | Thank you for your concern. We have addressed your concern.  Figure 1 shows the sample distribution in the 8 countries of south and south-east Asia. Since the DHS follows a complex sampling design (stratification, clustering, and weights) within the country, sampling weight were important in descriptive analysis. So, the authors applied the sampling weight after creating a pooled dataset of 8 countries using stata command svy and presented the result in table 1 (weighted percentage). Please see table 1, it is now re-written as weighted percentage instead of only percentage written earlier.  In the paper, the authors have mentioned that “STATA command “svy set” was used for this analysis to address the complex survey design” which is consistent with earlier published literature[1]. | Please refer to table 1  Page 9 |
| 2. | It is not clear how weighting was done in the multilevel analysis. | A sincere thanks for raising this issue. Sample weighting is important in descriptive analysis and precesion measurement to correct the standard error and confidence interval which we used in table 1 to calculate weighted percentage which is important for prevalence analysis. Since, by accounting for the data structure, multi-level modelling correct the standard error , it is unnecessary to use weight in multi-level analysis. Earlier literature also support this, for example, Angus deaton mentioned that in behavoral model using sample weight is not important [2]. Moreover, Bayesian statistician, Andrew Gelman also supported the notion that weights are just unnecessary in regression analysis [3]. | N/A |
| 3. | It is not clear how many levels of multilevel analysis were performed. Please mention in the statistical analysis section. | Thank you very much for your suggestion. We have addressed the issue. The levels are now mentioned in the analysis section as follows:  “This study considered two-level multi-level analysis: level 1 and 2 indicated individual level and country level, respectively..” | Changes are highlighted in red coloured text in the revised manuscript. Please refer to the “estimation strategies” section  Page 8, lines 6-7 |
| 4. | This study conclusion and recommendation not consist with study findings. In the conclusion this study mentions about antenatal care visit but didn’t use the variable in the analysis. | Thank you for highlighting this issue. We now have addressed the issue. We have omitted antenatal care visit from the conclusion. Abstract has also been adjusted to reflect the manuscript. The following statement has been included, “… obese women should deliver at a centre offering services by specialist obstetrics to ensure early neonatal care.” | Changes are highlighted in red coloured text in the revised manuscript. Please refer to the “conclusion” section (Page 19 line 10-13) and Abstract (Page 2 line 22-23). |

**Refernence:**

1.	Tesema GA, Yeshaw Y, Worku MG, Tessema ZT, Teshale AB. Pooled prevalence and associated factors of chronic undernutrition among under-five children in East Africa: A multilevel analysis. PLoS One. 2021;16: 1–17. doi:10.1371/journal.pone.0248637

2.	Deaton A. The Analysis of Household Surveys: A Microeconometric Approach to Development Policy. Reissue Ed. Washington, DC: World Bank: World Bank Publications; 2018. doi:10.1596/0-8018-5254-4

3.	Gelman A. Struggles with survey weighting and regression modeling. Stat Sci. 2007;22: 153–164. doi:10.1214/088342306000000691
